# Design and Optimization Principles of Cylindrical Sliding Triboelectric Nanogenerators

**DOI:** 10.3390/mi12050567

**Published:** 2021-05-17

**Authors:** Zhike Xia, Pei-Yong Feng, Xin Jing, Heng Li, Hao-Yang Mi, Yuejun Liu

**Affiliations:** 1 Key Laboratory of Advanced Packaging Materials and Technology of Hunan Province, Hunan University of Technology, Zhuzhou 412007, China; xiazhike@126.com (Z.X.); fpyedu@163.com (P.-Y.F.); yjliu_2005@126.com (Y.L.); 2 Department of Building and Real Estate, Hong Kong Polytechnic University, Hong Kong 518000, China; heng.li@polyu.edu.hk; 3 National Engineering Research Center for Advanced Polymer Processing Technology, Zhengzhou University, Zhengzhou 450003, China

**Keywords:** triboelectric nanogenerator, energy harvesting, sliding mode, spring parameter

## Abstract

Reciprocating motion is a widely existing form of mechanical motion in the natural environment. Triboelectric nanogenerators (TENGs) that work in sliding mode are ideal for harnessing large-distance reciprocating motion, and their energy conversion efficiency could be greatly enhanced by adding springs to them. Herein, we focused on investigating the design and optimization principles of sliding mode TENGs by analyzing the effects of spring parameters and vibration frequency on the triboelectric output performance of typical cylindrical sliding TENGs (CS-TENGs). Experimental study and finite elemental analysis were carried out based on a CS-TENG model assembled using a polytetrafluoroethylene (PTFE) film as the negative layer and an aluminum film as the positive layer. The energy output was found to be mainly affected by the change of relative displacement between the two friction layers, rather than the reactive force applied by the springs or the velocity of the sliding motion. However, the frequency of the output signals could be improved when the stiffness coefficient of the springs and the CS-TENG vibration frequency were increased. This study provides valuable directions for the design and optimization of sliding mode TENGs containing springs, and will motivate in-depth research on the fundamental principles of TENG operation.

## 1. Introduction

The main problem related to the energy crisis is the worldwide growing demand for natural resources to power industrial societies. Green energy harvesting technology has become a hot topic due to the fuel shortages and the environmental problems caused by the use of fossil-fuel-based energy [1,2]. Recently, with the rapid development of portable devices, harnessing environmental mechanical energy by using small devices is recognized as one of the new green energy sources of the era, and has attracted tremendous attention [3,4,5,6,7]. Such devices are usually called energy nanogenerators [8,9]. Nanogenerators based on different mechanisms—including piezoelectric effects, [10,11,12] electrostatic effects [13,14], and electromagnetic effects [15,16]—have been developed. Meanwhile, nanogenerators based on triboelectric effects have provided a new paradigm for developing cost-effective, high-output, flexible, and durable portable energy harvesters [17].

The triboelectric nanogenerator (TENG) is based on the coupled effect of triboelectrification and electrostatic induction, and is a new application of Maxwell’s displacement current in energy harvesting [18]. It can be designed in various forms using a wide range of materials, and has shown a powerful capability to harvest energy from almost all kinds of mechanical motion, such as random vibrations [19], wind flow [20], ocean waves [21], air pressure [22], and body movement [23]. 

There are essentially four working modes of TENGs, including vertical contact-separation mode, linear sliding mode, single-electrode mode, and freestanding triboelectric-layer mode [24]. Among them, the vertical contact-separation mode has been investigated most, while the linear sliding mode has unique advantages in collecting energy generated by long-distance reciprocating motion because of its excellent structure design and stable output performance [25]. In order to adapt the reciprocating motion, springs or rubber bands are often used in sliding mode TENGs. Springs and rubber bands were used in recent studies to store elastic potential by constructing a pendulum-like structure, which could reduce energy loss during the TENG’s operation so as to realize continuous swing after mechanical triggering, and amplify the operation frequency while enhancing the energy conversion efficiency [26,27,28]. However, the majority of studies have focused on the assembly, materials, and application of the TENGs [29,30,31,32,33]. The effects of spring properties and the optimization principle of the spring-based sliding mode TENG were rarely reported.

In this work, cylindrical sliding TENGs (CS-TENGs) were developed as a typical model in order to investigate the pivotal factors and the optimization principles of sliding mode TENGs equipped with springs. The TENG was designed to vibrate periodically in a vertical direction in order to convert mechanical energy into electrical energy. The inner cylinder and the outer tube, moving in coaxial directions, were assembled, together with a polytetrafluoroethylene (PTFE) film that served as the negative layer and an aluminum film that served as the positive layer. The motion behavior of the CS-TENG devices was simulated by using the finite element analysis method to investigate the effects of the springs’ stiffness coefficient and the vibration frequency on the triboelectric output performance. The working mechanism was elaborated in detail and validated by the experimental results. In addition, the optimized vibration state and the dependence of the output on the relative displacement of the CS-TENG were obtained from the experiments and verified by the simulation.

## 2. Materials and Methods

### 2.1. Anode Oxidization of Aluminum Film

A piece of aluminum film with dimensions of 60 mm × 120 mm × 0.4 mm was treated with the anode oxidation (AO) process in order to increase the surface roughness of the film, in accordance with the literature [9]. Briefly, the aluminum film was placed in a mixed solution of oxalic acid (30 g/L) and citric acid (20 g/L) in a beaker with a platinum electrode. The aluminum film was used as the anode and the platinum was used as the cathode. The AO process was conducted at 30 V for 1 h, and then the aluminum film was removed from the bath and rinsed with water and ethanol, followed by drying with nitrogen gas.

### 2.2. Fabrication of CS-TENGs

The CS-TENGs were composed of a solid aluminum cylinder with a diameter of 32 mm and a height of 50 mm, and a hollow polypropylene tube with an inner diameter of 34 mm and a length of 100 mm. A polytetrafluoroethylene (PTFE) film with a length of 50 mm was stuck around the aluminum cylinder, and a piece of AO-treated aluminum film with a length of 50 mm was pasted onto the inner surface of the PP cylinder. Current leads were connected to the aluminum cylinder and the aluminum film in order to direct the current flow to the external circuit. Different numbers of springs were glued onto PMMA plates, and the PMMA plates were then glued onto the two ends of the outer tube in order to enclose the inner cylinder inside the tube. The inner cylinder could thus move freely in the device, and be bounced back once it encountered the springs. In the meantime, the PTFE film and the aluminum film had a close distance of about 0.3 mm, allowing for friction between the two layers while the CS-TENGs were in operation.

### 2.3. CS-TENG Performance Evaluation

The CS-TENGs were operated in a vertical direction at different frequencies using a rocker arm. The output voltage signal generated by the CS-TENGs was recorded using an oscilloscope (Rigol, ZDS3034 Plus, Zhiyuan Corp., Guangzhou, China). The output current signal generated by the CS-TENGs was recorded via a potentiostat (CHI760E), using the i-t method. The CS-TENGs were firstly operated for 1 min prior to data acquisition, in order to ensure that they reached a stable output state.

### 2.4. Finite Element Analysis Using ANSYS

ANSYS software was used to simulate the motion process of the CS-TENG devices. A virtual model with the same geometry and unit dimensions as the CS-TENG devices was established. The quality, material, and movement function of the virtual model was defined. The motion form of the virtual model was set as reciprocating motion, in order to conform to the actual situation. The stiffness coefficient of the springs and the vibration frequency of the device were changed in the simulation in order to investigate their effects on the motion behavior of the inner cylinder relative to the outer tube.

For the simulation process, the virtual model was meshed, and the complex model was divided into several small parts in order to improve the calculation accuracy. An example of the motion behavior simulated is shown in Video S1. By analyzing the function graph of the virtual model, the total displacement and the relative displacement curves of the aluminum electrode and the PTFE film were obtained.

### 2.5. COMSOL Multiphysics Simulation

The potential distribution of the CS-TENG was simulated using COMSOL Multiphysics software based on the electrostatic (ES) function. Two-dimensional models were created to reflect both the fully matched situation and the mismatched situation of the PTFE film and the aluminum film. The positive and negative layers were defined based on the dielectric constants of PTFE and aluminum, respectively. The surrounding environment was set as air. The steady-state potential distributions of the CS-TENG in different states were simulated in order to investigate the positive and negative electrostatic field distribution.

## 3. Results and Discussion

**Figure 1:**
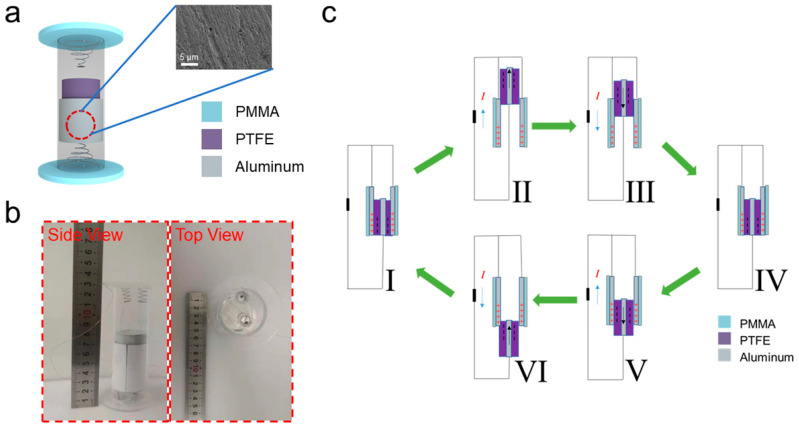
(**a**) Schematic Illustration of the CS-TENG device assembled using a PP outer tube attached to an AO-treated aluminum film, an aluminum inner cylinder wrapped in a PTFE film, and two springs fixed on each end of the device, which was enclosed by two PMMA plates. Inset image shows the rough surface morphology of the AO-treated aluminum film. (**b**) Digital photographs of the CS-TENG taken from the top view and the side view. (**c**) Schematic diagram showing the working principles of the developed CS-TENG, divided into six basic stages.

The structural configuration of the CS-TENG is depicted in Figure 1a. An inner cylinder was coaxially assembled with an outer tube, and the inner cylinder could slide freely within the tube. Two springs were fixed on both ends of the outer tube so that the inner cylinder could be bounced back and forth when the CS-TENG was in operation. AO-treated aluminum film was stuck onto the inner wall of the outer polypropylene tube, and served as the positive electrode. The AO treatment could increase the roughness of the aluminum film effectively, as indicated by the inset SEM image in Figure 1a. The AO approach has been widely used in previous studies to modify aluminum foils [34,35,36]. PTFE film was pasted onto the outer wall of the inner aluminum cylinder, and served as the negative electrode. The working principle of the CS-TENG was based on sliding triboelectrification at the interface between the PTFE layer and aluminum layer. The inner diameter of the outer tube was 34 mm, the outer diameter of the inner cylinder was 32 mm, the thickness of the PTFE film was 0.2 mm, and the thickness of the aluminum film was 0.4 mm. Thus, the two layers were controlled to have a tiny gap (about 0.4 mm), in order to allow the cylinders to move freely in a coaxial 1D motion and still be able to abrase with one another during the movement. Two copper wires were connected to the inner cylinder and the aluminum film was attached to the outer tube as current leads, in order to direct the generated current to the outer circuit. Figure 1b shows the overall size and appearance of the assembled CS-TENG device from the side view and the top view.

Figure 1c illustrates the working mechanism of the CS-TENG device and its connection to the external circuit. The whole process can be divided into six stages. In the neutral state, the PTFE film completely overlaps with the aluminum film, and opposite charges would generate due to the coupling effects of contact electrification and electrostatic induction. An equal amount of positive and negative charges would be generated on the aluminum film and the PTFE film, respectively (Figure 1c-I). As the device moved downward, the two surfaces of the cylinder and the tube would slide under external mechanical excitation, and a potential difference would form accordingly, which triggered the flow of electrons from the inner aluminum cylinder stuck with the PTFE film to the aluminum film attached to the inner surface of the outer tube; thereby, a forward current flow was formed in the external circuit (Figure 1c-II). When the inner aluminum cylinder was bounced back by the spring, the mismatched area would become smaller as the inner cylinder slid towards the center of the outer tube. This process created another current flow in the opposite direction in the external circuit (Figure 1c-III). After a short neutral state (Figure 1c-IV), the inner aluminum cylinder would slide to the other end of the outer tube, and the PTFE film would move away from the aluminum film, creating another current flow from the inner aluminum cylinder to the aluminum film in the external circuit (Figure 1c-V). Similarly, when the inner cylinder was bounced back by the bottom spring, an opposite current flow would be generated (Figure 1c-VI). Through a series of vibrational processes, the developed CS-TENG could repetitively convert the mechanical energy to alternating current (AC) energy by creating a current flow in the circuit between the two electrodes.

So far various TENGs working in the sliding mode have been developed. Most of them work in the rotation mode [8], but some are based on the reciprocating mode [37]. Springs and rubber bands have been used in the design of these sliding TENGs [38,39]. However, there is no literature focused on investigating the motion behavior and the effects of spring properties on the performance of reciprocating sliding TENGs. This study mainly focused on revealing the motion behavior of the two friction materials and the effect of the spring stiffness coefficient on the energy output performance of a typical cylindrical sliding TENG (CS-TENG), as developed above.

**Figure 2:**
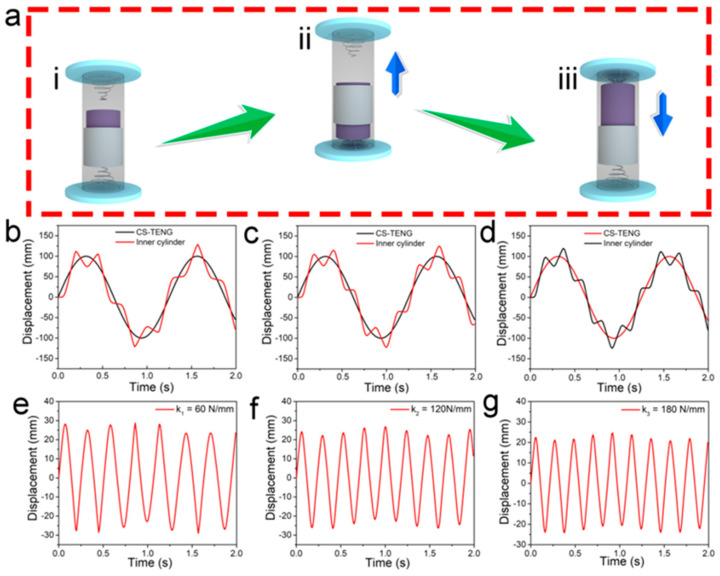
(**a**) Illustration of the CS-TENG’s movement during operation, reflecting the relative position of the inner cylinder to the outer tube. Finite element analysis of the CS-TENG using ANSYS: (**b**–**d**) motion trajectories of the CS-TENG device and the inner cylinder, and (**e**–**g**) the normalized displacement of the inner cylinder when using springs with stiffness coefficients of k_1_ = 60 N/mm, k_2_ = 120 N/mm, and k_3_ = 180 N/mm, respectively.

Intuitively, the energy generated by the CS-TENG is related to the relative displacement of the two electrodes when the whole device undergoes reciprocated motion. As illustrated in Figure 2a, the movement of the inner cylinder had a lag compared with the outer tube due to inertia. The springs fixed on the two ends would give a counterforce to the inner cylinder to accelerate its motion. As expected, the movement and the mismatched area of the inner cylinder relative to the outer tube greatly affected the performance of the whole CS-TENG device. Given a certain input power, the stiffness coefficient of the spring was a critical factor determining the movement of the inner cylinder relative to the outer tube and the triboelectric output performance. A 3D model of the CS-TENG device was established using ANSYS software to perform a 3D finite element simulation. The geometry, size, and mass of each component were modeled to match the developed CS-TENG, and three different spring stiffness coefficients (k1 = 60 N/mm, k2 = 120 N/mm, and k3 = 180 N/mm) were used in the simulation. Since TENG devices generally work in a simple harmonic motion, a motion function *f*_(*x*)_ = *Asin*(2*πωt*) (*A* = 100, *f* = 0.5) was used as the input function in order to mimic the movement of the CS-TENG device. The motion direction of the inner cylinder was restricted to the same direction as the whole device, in order to reflect the confined environment in the real case. The motion trails of the inner cylinder and the outer tube over time could be simulated after meshing and running the calculation.

The motion trajectories (displacements) of the CS-TENG device and the inner cylinder are shown in Figure 2b–d for the use of springs with different stiffness coefficients. The simulated results revealed that the inner cylinder, which was wrapped in the PTFE film, exhibited an oscillating behavior, while the whole device underwent a sinusoidal motion. It was found that the overall displacement of the whole device remained the same when using different springs, but the reciprocating motion frequency of the inner cylinder was gradually increased as the stiffness coefficient of the springs was enhanced from 60 N/mm to 180 N/mm. By normalizing the displacement of the inner cylinder with the displacement of the whole device, it was easier to observe the changing trends of the inner cylinder as the stiffness coefficient of the springs was increased. As shown in Figure 2d–f, the inner cylinder showed a periodic movement pattern with little fluctuation. The normalized displacement represents the distance between the center of gravity of the inner cylinder and the outer tube. It was found that this displacement gradually decreased with the increase of the stiffness coefficient of the springs. This was because greater counterforce was exerted on the inner cylinder when it impacted on a stiffer spring with a certain momentum, leading to smaller compression of the spring and a greater adverse acceleration speed. Therefore, stiffer springs in the CS-TENG would cause a faster motion but a shorter motion distance to the inner cylinder. It is believed that this movement phenomenon would greatly affect the triboelectric output performance of the CS-TENGs. The following section provides a concise and precise description of the experimental results, their interpretation, and the experimental conclusions that can be drawn from them.

**Figure 3:**
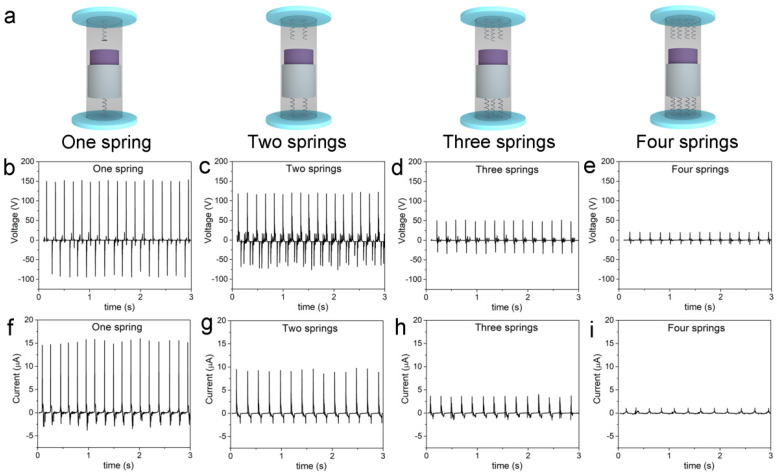
(**a**) Prototype designs of the CS-TENGs with different numbers of springs, reflecting the difference in stiffness coefficient. (**b**–**e**) Output voltage results of CS-TENGs with 1–4 springs fixed on each end of the device. (**f**–**i**) Output current results of CS-TENGs with 1–4 springs fixed on each end of the device.

In the experiment, we were able to maintain the motion of the CS-TENG in a vertical direction, so that the inner cylinder could maintain a 1D coaxial motion relative to the outer tube. In order to investigate the effect of the stiffness coefficient of the springs, we assembled four CS-TENGs equipped with different numbers of springs on each end of the device, as illustrated in Figure 3a. The stiffness coefficient of the springs was calculated to be 4.8 N/mm, and the stiffness coefficient would be multiplied by the increase of the number of springs from 1 to 4. The CS-TENGs were moved periodically in a vertical direction until stable signals were obtained, and the output voltage and current signals were recorded using an oscilloscope and an electrochemical working station. Figure 3b–e shows the output voltage of 4 CS-TENGs when they were operated vertically at a frequency of 4 Hz. Although all of the CS-TENGs could produce stable electrical output, under the same excitation, their output voltage displayed significant differences, and the voltage demonstrated an increasing trend as the stiffness coefficient of the springs in the TENGs was increased. The output current results (Figure 3f–i) showed the same trend as the output voltage results.

**Figure 4:**
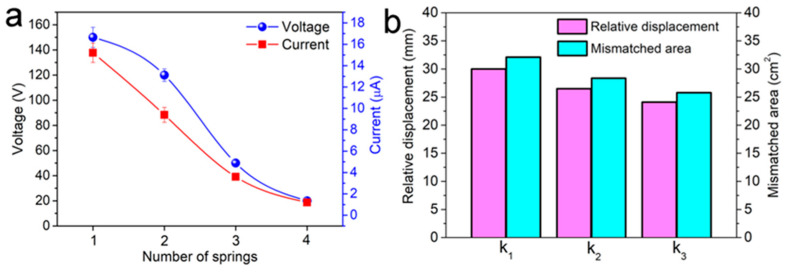
(**a**) Output voltage and current statistical results for the CS-TENGs with different numbers of springs fixed on each end. (**b**) Simulation results for the maximum relative displacement and the mismatched area between the inner cylinder and the outer tube for the CS-TENGs equipped with springs with increased stiffness coefficient.

The output voltage and current statistical results of four CS-TENGs are compared in Figure 4a, from which it is clear that the output voltage and current for the CS-TENG equipped with one spring on each end were the highest, at 150 V and 15 µA, respectively. As the number of springs increased, the output power of the CS-TENGs rapidly decreased. When the number of springs reached 4, the output voltage and current dropped to 20 V and 1.2 µA, respectively. This is because the stiffness coefficient of the springs increased in the form of superposition when the springs were arranged in a parallel state. As verified by the simulation results, the compression deformation of the springs by the inner cylinder was smaller when there were more springs, while the frequency and vibration amplitude remained constant. This resulted in a decrease in the relative displacement between the inner cylinder (negative PTFE film) and the outer tube (positive aluminum film), which was reflected by the shift in the center of gravity to the inner cylinder from the outer tube, as shown in Figure 4b. The relative displacement between the inner cylinder and the outer tube during the CS-TENGs’ operation caused changes to the mismatched area between the PTFE film and the aluminum film, which was the main cause of the generation of current flow in the external circuit. The mismatched area was also gradually reduced as the spring stiffness coefficient increased, explaining the decreased output performance as the number of springs assembled in the CS-TENG was increased.

**Figure 5:**
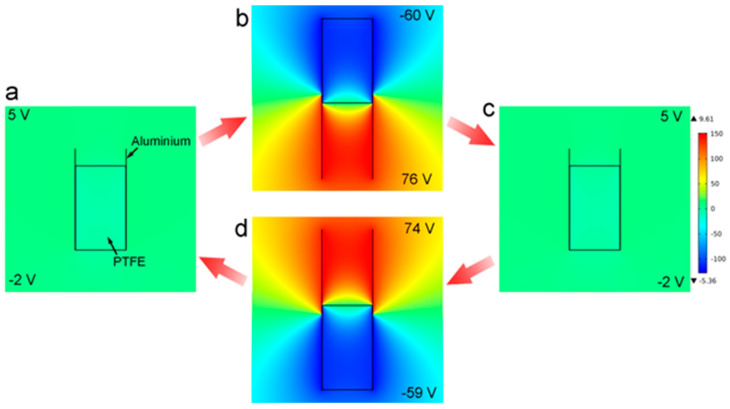
COMSOL Multiphysics simulation results for the CS-TENGs in different operating states. (**a**) The starting position. (**b**) The process of upward movement. (**c**) The starting position. (**d**) The downward motion process.

In addition, COMSOL Multiphysics software was used to simulate the potential field distribution of the CS-TENGs in different operating states. As shown in Figure 5, the potential difference between the top region and the bottom region was only 7 V when the CS-TENG devices were in the matched state (Figure 5a). Although the electrostatic field was insignificant, charge transfer occurred when the aluminum film was in contact with the PTFE film. Hence, a great potential difference could be generated when the inner cylinder and the outer tube moved away from one another. It was found that a high voltage difference of 136 V could be generated when the PTFE film attached to the inner cylinder was moved upward such that it departed from the aluminum film stuck to the inner surface of the outer tube (Figure 5b). The CS-TENG devices would adopt a relatively neutral state when the inner cylinder moved back to the matched state (Figure 5c), and the electrostatic field would be reversed when the inner cylinder was moved downward beneath the aluminum film (Figure 5d). The simulation results verified that a great electrostatic field would be created periodically as the inner cylinder slid up and down in the region confined by the outer tube, due to the triboelectric difference between the PTFE film and the aluminum film attached to the inner cylinder and the outer tube, respectively. 

**Figure 6:**
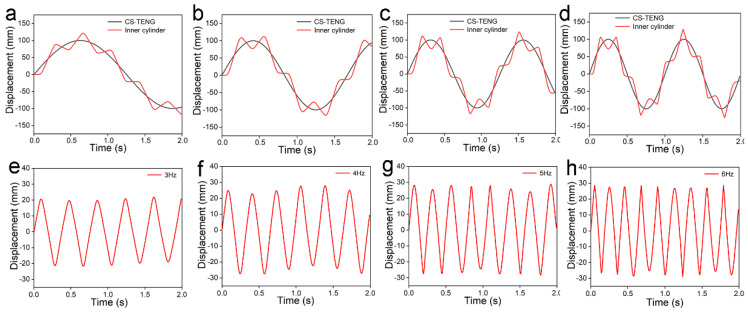
Finite element analysis of the CS-TENGs using ANSYS: (**a**–**d**) motion trajectories of the CS-TENG devices and the inner cylinder, and (**e**–**h**) the normalized displacement of the inner cylinder when the CS-TENGs were operated under frequencies of 3 Hz, 4 Hz, 5 Hz, and 6 Hz, respectively.

One of the most important external factors in the CS-TENGs’ operation is the vibration frequency of the devices, while the effect of sliding mode TENGs’ vibration frequencies on their energy output performance is not yet fully elaborated. In this study, the CS-TENGs’ operation under different frequencies was also simulated using ANSYS finite element analysis. In the simulation, the motion frequency of the virtual model was set at 3 Hz, 4 Hz, 5 Hz, and 6 Hz; the motion amplitude was fixed for all cases, and the total displacement and the relative displacement results were analyzed accordingly. As shown in Figure 6a–c, it was clear that the motion frequency of both the CS-TENG devices and the inner cylinder was greatly enhanced with the increase of frequency from 3 Hz to 5 Hz. Interestingly, it was found that although the displacement of the CS-TENGs was fixed at 100 mm for all cases, the relative displacement between the aluminum film and PTFE film gradually increased as the operating frequency increased (Figure 6e–g). However, when the motion frequency was increased to 6 Hz, the relative displacement between the aluminum film and the PTFE film was approximately the same as that at 5Hz, while the motion frequency of the inner cylinder was clearly increased (Figure 6h). This implies that the compression degree of the springs might have reached its maximum level when the vibration frequency of the CS-TENGs exceeded 5 Hz. Therefore, further increases in vibration frequency would not enhance the relative displacement, but would increase the motion frequency and velocity of the inner cylinder.

**Figure 7:**
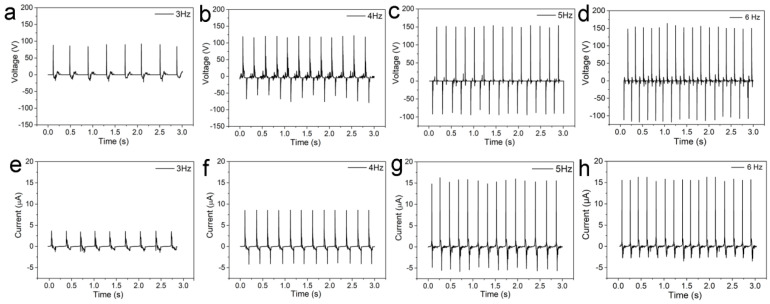
(**a**–**d**) Output voltage results for CS-TENGs when operated vertically at a vibration frequency of 3–6 Hz. (**e**–**h**) Output current results for CS-TENGs when operated vertically at a vibration frequency of 3–6 Hz.

The influence of the vibration frequency of the CS-TENG devices on their output performance was studied by vertically shaking the CS-TENGs at different frequencies of 3 Hz, 4 Hz, 5 Hz, and 6 Hz, at a constant vibration amplitude of 100 mm. The output voltage and current results were measured and compared, as shown in Figure 7. It was found that, as the vibration frequency increased, both the voltage signal frequency and the output voltage (Figure 7a–c) increased significantly. The output current signal (Figure 7e–f) showed the same trend. These results indicate that the CS-TENGs can generate a stable output signal under various frequencies, and that the operating frequency is highly related to the energy output of the CS-TENGs. In addition, we found that the voltage and current signals could not be further enhanced when the voltage reached over 150 V, even when the vibration frequency was further increased. This was due to the fact that the relative displacement between the aluminum film and the PTFE film almost reached the maximum distance because of the size limitation of the CS-TENG devices. The PTFE film and the aluminum film reached their maximum relative displacement, beyond which no significant changes in the charge transfer and the potential difference would occur.

**Figure 8:**
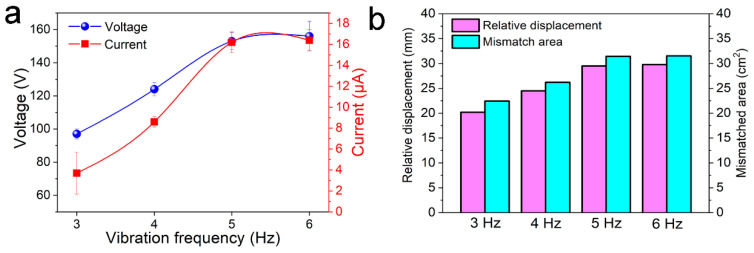
(**a**) Output voltage and current statistical results for the CS-TENGs operated vertically at a vibration frequency of 3–6 Hz. (**b**) Simulation results for the maximum relative displacement and the mismatched area between the inner cylinder and the outer tube for the CS-TENGs operated vertically at vibration frequency of 3–6 Hz.

For easy comparison, the statistical data is plotted in Figure 8a. We found that the output voltage increased from 97 V to 153 V and the output current increased from 3.7 μA to 16.2 μA when the vibration frequency was enhanced from 3 Hz to 5 Hz. The voltage and current remained at the same levels when the vibration frequency was increased from 5 Hz to 6 Hz. The finite element simulation results (Figure 8b) revealed that the relative displacement and the mismatched area between the inner cylinder and the outer tube increased when the frequency was increased from 3 Hz to 5 Hz, while it remained about the same when the frequency was further enhanced to 6 Hz, which is consistent the experimental results. These results demonstrate that the peak output voltage and current of the CS-TENGs were mainly affected by the relative displacement between the negative PTFE film and the positive aluminum film, rather than the frequency or the motion velocity. However, the energy generation frequency could be significantly increased with the increase in operating frequency, due to the reactive force applied by the springs. Therefore, the results and basic conclusions obtained from this study may provide valuable directions for the design of sliding mode TENGs containing springs, as well as guidance for the optimization of their output performance.

## 4. Conclusions

In this work, we developed a typical CS-TENG working in one-dimensional sliding mode and focused on investigating the effects of the stiffness coefficient of the springs, and the vibration frequency, on the output performance of the CS-TENG. Electrostatic potential simulation results revealed that the relative displacement between the PTFE film attached to the inner cylinder and the aluminum film stuck to the inner surface of the outer tube was the main cause of the potential difference and the current flow in the external circuit. The springs fixed on both ends of the CS-TENG played a pivotal role in affecting the bouncing frequency and motion distance of the inner cylinder. Springs with a lower stiffness coefficient resulted in higher output because of their greater relative displacement between the PTFE film and the aluminum film. In addition, the output energy was increased when the operation frequency was enhanced. However, the improvement became insignificant when the frequency exceeded 5 Hz, indicating that an optimal level could be reached. In addition, we found that the peak output voltage could not be enhanced by further improving the sliding speed, but the increase in the device’s vibration frequency could greatly enhance the frequency of the energy output. This study revealed critical factors influencing the output performance of sliding mode TENGs, and offers design and optimization principles for sliding mode TENGs containing springs.

## Data Availability

The data presented are available on request from the corresponding authors.

## Supplementary Materials

The following are available online at https://www.mdpi.com/article/10.3390/mi12050567/s1, Video S1: Animation of the finite elemental analysis of the CS-TENG.
